# Pathophysiologic implications and therapeutic potentials of telocytes in multiorgan fibrosis

**DOI:** 10.1097/BOR.0000000000001116

**Published:** 2025-08-01

**Authors:** Irene Rosa, Eloisa Romano, Bianca Saveria Fioretto, Mirko Manetti

**Affiliations:** Department of Experimental and Clinical Medicine, University of Florence, Florence, Italy

**Keywords:** antifibrotic therapy, intercellular signaling, multiorgan fibrosis, telocytes, tissue homeostasis

## Abstract

**Purpose of review:**

Telocytes (TCs) are unique stromal cells with distinctive morphology, ultrastructural features, and intercellular communication abilities. Accumulating evidence supports their critical roles in tissue homeostasis, regeneration, and stem cell niche maintenance through both cell-to-cell contacts and delivery of paracrine signals. The purpose of this review is to provide an up-to-date overview of the current knowledge regarding the pathophysiologic implications and therapeutic potentials of TCs in multiorgan fibrosis.

**Recent findings:**

Loss and/or structural degeneration of TCs have been implicated in the pathogenesis of fibrotic conditions affecting the skin, gastrointestinal tract, heart, lungs, kidneys, and reproductive organs. TC depletion has often been associated with extracellular matrix remodeling, aberrant fibroblast activation, disruption of stem cell support, and altered tissue architecture. Experimental evidence suggests that TCs may possess antifibrotic therapeutic potentials, with TC transplantation or administration of TC-derived secretome/extracellular vesicles mitigating fibrosis progression in different preclinical models.

**Summary:**

TCs are emerging as pivotal regulators of stromal homeostasis across several organs and their loss appears to be a unifying feature in the pathogenesis of tissue fibrosis in different anatomical districts. Targeting TCs, either by preserving their function or restoring their networks/paracrine signals, may open new therapeutic avenues for managing various fibrotic diseases.

## INTRODUCTION

Telocytes (TCs) represent a distinct population of interstitial cells ubiquitously distributed within the stromal compartment of numerous organs and characterized by unique morphologic, ultrastructural, and functional properties [1–6]. TCs are easily identifiable by their small piriform, spindle or triangular cell body and by their extremely thin, long, and branched telopodes, characteristic cytoplasmic processes with a typical moniliform aspect due to the alternation of very slim segments (podomers) and small enlarged portions (podoms) [1–6]. Within tissues, telopodes are usually organized in intricate 3D networks, which makes them highly specialized for both homocellular and heterocellular communications [1–6]. Moreover, telopodes can also establish contacts with the extracellular matrix (ECM) [7]. Under transmission electron microscopy (TEM), which is considered the gold standard technique to observe TCs, these stromal cells are characterized by a relatively small cell body containing scarce perinuclear cytoplasm, with mitochondria and endoplasmic reticulum cisternae mainly harbored within podoms [1–6]. In addition, telopodes are frequently surrounded by various types of extracellular vesicles, including exosomes, ectosomes, and multivesicular bodies, supporting a role for TCs in paracrine signaling and local cellular regulation [7,8,9^▪▪^]. In terms of immunophenotype, although TCs lack a distinctive antigenic profile, expression of CD34 alone or in combination with platelet-derived growth factor receptor α (PDGFRα) currently represents the most reliable label for their identification under light microscopy [1–6]. Nevertheless, the specific TC immunophenotype may vary across different tissue or organ systems, and heterogeneous TC subpopulations exhibiting distinct immunohistochemical features may coexist within the stroma of a single anatomical site [4,7,10,11]. Beyond these ultrastructural and immunophenotypic features, emerging data reveal that TCs also possess unique microRNA signatures and gene/protein expression profiles, which clearly distinguish them from classical fibroblasts and other stromal cell types [4,7,10,11]. While the full spectrum of TC functions remains to be fully elucidated, their characteristic spatial organization, extensive cell-to-cell communications, and paracrine activity via extracellular vesicle release suggest that these peculiar stromal cells may play significant roles in a wide range of physiological processes [2,4,7,10,11]. First, by forming 3D labyrinthine networks with their long and interconnecting telopodes, TCs may serve as a structural scaffold, guiding tissue morphogenesis, promoting postnatal repair, and maintaining tissue integrity [2,4,7,10,11]. Second, TCs have been proposed to mediate cellular signaling both via direct cell-to-cell contacts and indirectly through vesicle-mediated delivery of cytokines, growth factors, and RNAs (e.g., mRNAs, microRNAs and other noncoding RNAs) [2,4,7,10,11]. Third, TCs were also identified as an emerging component of stem cell niches of several organs, where they are thought to be crucial for stem cell survival, renewal, differentiation, maturation, and guidance [2,4,7,10,11]. Lastly, TCs are commonly regarded as active participants in immunomodulation/immunosurveillance, electrical signal conduction especially in the myometrium and myocardium, and regulation of intestinal motility, likely through the diffusion of the slow waves generated by the interstitial cells of Cajal within the enteric neuromuscular compartment [2,4,7,10,11].

**FIGURE 1:**
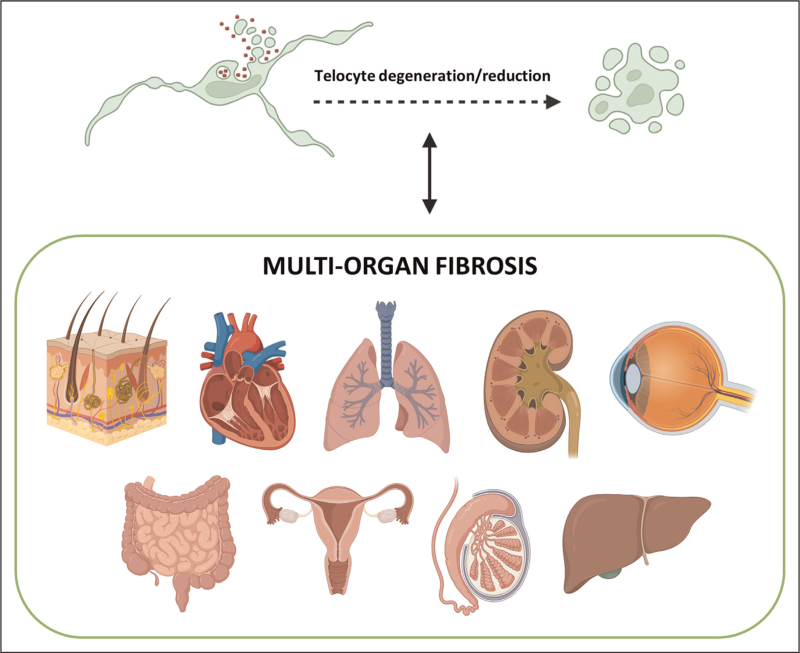
Degeneration and reduction of telocytes, a distinctive stromal cell population possessing unique morphologic features and intercellular communication abilities, have been associated with the onset and progression of multiorgan fibrosis, including the skin, heart, lungs, gastrointestinal tract, kidneys, liver, organs of the reproductive systems, and cornea. Telocyte damage or loss may either be a consequence of the fibrotic process or precede the initial stages of fibrosis, and a reciprocal causation between fibrosis establishment and telocyte impairment may exist.

Given their broad distribution and multifaceted functions, increasing interest has focused on the involvement of TCs in pathologic conditions, particularly fibrotic diseases, which are characterized by excessive ECM deposition and remain a challenging clinical problem due to their unclear pathogenesis and limited therapeutic options [4,6,7,10–14]. In this context, a growing body of evidence has recently established a strong association between TC morphologic and numerical impairment and the progression of a wide range of disorders featuring tissue fibrosis, including systemic sclerosis (SSc or scleroderma), ulcerative colitis (UC), Crohn's diseases (CD), liver fibrosis, myocardial fibrosis, and endometriosis among others (Fig. 1) [4,6,7,10–14]. Nonetheless, it should be considered that TC damage and loss may either be a consequence of the fibrotic process or precede the initial stages of fibrosis, and that a reciprocal causation between fibrosis establishment and TC impairment cannot be excluded (Fig. 1). However, since accumulating literature reported that TC transplantation and/or TC-derived secretome/extracellular vesicle administration were able to mitigate ECM deposition in preclinical models of several fibrotic diseases, TCs may be regarded as a promising innovative antifibrotic therapeutic tool [7,12,15^▪^,16^▪^,^17^,^18^].

In the present review, we will provide a comprehensive overview of the most important findings regarding TC involvement in different fibrotic conditions, and critically examine TC therapeutic potential for the management of these challenging pathologies. 

**Box 1:**
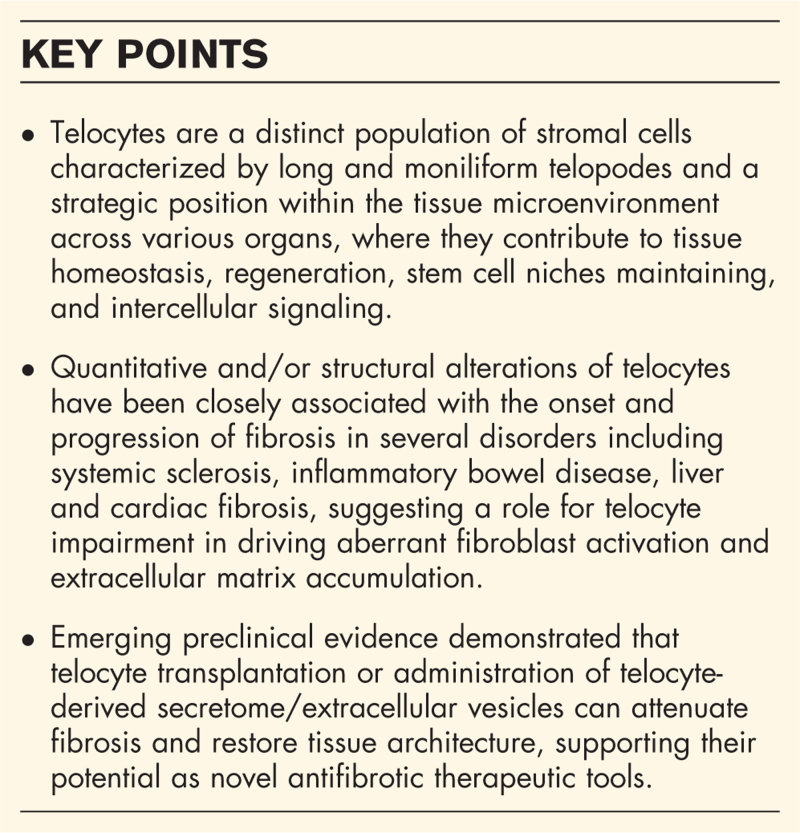
no caption available

## TELOCYTES IN SKIN FIBROSIS

**FIGURE 2:**
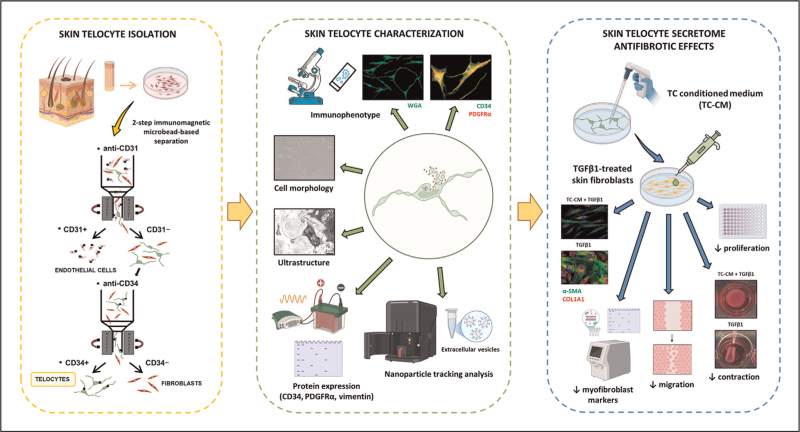
Skin telocyte isolation, characterization, and in vitro assessment of their antifibrotic effects. Skin telocytes were isolated from human healthy skin biopsies by using a two-step immunomagnetic microbead-based cell separation method, in which total dermal cells were subjected to magnetic-activated cell sorting: in the first step, anti-CD31-conjugated beads were used to isolate CD31+ endothelial cells that share CD34 expression with telocytes, while in the second step, performed on the remaining CD31− cells, anti-CD34-conjugated beads were used to further separate CD31−/CD34− fibroblasts from CD31−/CD34+ telocytes. Skin telocytes were subsequently cultured and characterized using different methodologies: (i) morphologic features were assessed via phase-contrast microscopy and wheat germ agglutinin (WGA) fluorescent staining of the plasma membrane; (ii) immunophenotypic profiling was performed by double immunofluorescence staining for CD34 and PDGFRα; (iii) CD34, PDGFRα, and vimentin protein expression was evaluated by immunoblotting; and (iv) extracellular vesicle content in telocyte conditioned medium (TC-CM) was analyzed using nanoparticle tracking analysis. Skin TC-CM exerted antifibrotic effects by effectively preventing transforming growth factor β1 (TGFβ1)-induced skin fibroblast-to-myofibroblast differentiation. Indeed, TC-CM significantly prevented the proliferative and migratory responses of skin fibroblasts to TGFβ1 stimulation, as well as their phenotypic differentiation into profibrotic and contractile COL1A1+/α-SMA+ myofibroblasts.

In healthy skin, TCs establish an extensive 3D interstitial network that compartmentalizes the dermal layer, with their telopodes closely surrounding microvascular structures, peripheral nerve fibers, and cutaneous adnexa, including hair follicles and sweat glands [19]. In the setting of cutaneous fibrosis, and particularly in SSc, a complex autoimmune connective tissue disorder characterized by microvasculopathy and progressive fibrosis of the skin and internal organs, remarkable alterations in the TC network have been documented [19]. Specifically, a significant reduction and degenerative changes in cell morphology of dermal TCs have been observed in fibrotic skin biopsies from SSc patients, correlating with the extent of dermal fibrosis and featuring ultrastructural abnormalities such as mitochondrial swelling, cytoplasmic vacuolization, and accumulation of lipofuscin bodies [19]. Similar findings have been replicated in the bleomycin-induced murine model of scleroderma-like dermal fibrosis [20]. Different hypotheses have been proposed to explain the relationship between TC damage/loss and the onset/progression of dermal fibrosis. First, TC damage/loss may either be a consequence of ECM remodeling during fibrogenesis or disrupt the regulatory role of TCs over fibroblasts, thus contributing to abnormal fibroblast activation and consequent differentiation into profibrotic myofibroblasts [7,9^▪▪^,^19^,^20^]. Second, TCs themselves might undergo a phenotypic conversion into myofibroblasts, thus directly contributing to the fibrotic stromal cell pool [7,13]. However, experimental evidence overwhelmingly supports the first hypothesis as the dominant mechanism. Indeed, while few cells co-expressing the TC marker CD34 and the myofibroblast marker α-smooth muscle actin (α-SMA) have been detected in fibrotic SSc skin and in bleomycin-treated mouse skin at early stages of the fibrogenic process [13,20], in dermal lesions of both SSc patients and bleomycin-induced mouse model TEM analysis provided clear evidence of progressive degenerative ultrastructural changes and necrosis of TCs rather than their transdifferentiation into profibrotic myofibroblasts [19,20]. In addition, a recent clinical case study by Pereira de Godoy *et al.* described pronounced dystrophic changes in dermal TCs (i.e., telopode fragmentation, cytoplasmic disintegration, apoptotic nuclear morphology, and nuclear extrusion) during the progression of lower limb lymphedema-associated fibrosis [21]. Notably, intensive lymphatic stimulation therapy resulted in a marked increase in TC density and was associated with clinical reversal of dermal fibrosis [21]. Finally, recent in vitro findings highlighted important antifibrotic properties of skin TCs, which could be exploited in the near future to develop novel therapeutic strategies against cutaneous fibrosis [9^▪▪^]. In fact, through a combination of morphologic, gene/protein expression, and functional investigations, TC secretome as conditioned medium collected from cultured healthy dermal TCs isolated using a two-step immunomagnetic microbead-based protocol was shown to be rich in extracellular vesicles and effective in preventing transforming growth factor β1 (TGFβ1)-induced skin fibroblast-to-myofibroblast profibrotic differentiation (Fig. 2) [5,9^▪▪^]. Indeed, the administration of TC-conditioned medium not only significantly suppressed the proliferative, migratory, and contractile responses of fibroblasts to TGFβ1 stimulation, but also attenuated their profibrotic phenotypic differentiation into myofibroblasts, as evidenced by a marked decrease in *FAP*, *ACTA2*, *COL1A1*, *COL1A2*, *FN1*, and *CTGF* gene expression levels, along with reduced α-SMA, N-cadherin, COL1A1, and fibronectin containing extra domain A (FN-EDA) protein levels (Fig. 2) [9^▪▪^]. Collectively, these promising findings pave the way for further preclinical research to understand in-depth the skin TC-derived paracrine signals that are crucial in the control of fibroblast behavior and might be suitable as novel targets for antifibrotic therapies. Besides TC paracrine functions, whether transplantation of skin TCs directly within fibrotic skin lesions might represent an additional therapeutic option deserves thorough investigation.

## TELOCYTES IN GASTROINTESTINAL TRACT FIBROSIS

TCs are widely distributed across all the layers of the gastrointestinal tract wall, where they contribute to maintaining structural integrity and functional regulating the local microenvironment [7,10,11]. Within intestinal stem cell niches, particularly in the mucosal compartment, TCs play a pivotal role by delivering paracrine signals such as Wnt ligands, which are essential for stem cell maintenance/renewal and epithelial turnover [7,10,11,22,23]. In the context of inflammatory bowel disease (IBD), including CD and UC, two complex pathologies in which chronic relapsing and remitting intestinal inflammation finally results into extensive tissue fibrosis and consequent intestinal stiffness and dysmotility, TC morphology and distribution undergo significant pathologic alterations that are closely associated with the degree of fibrotic remodeling and structural disorganization of the intestinal wall [​​​​​​​^7^,^10^–^12^,^24^. In CD, inflammation and fibrosis may involve the entire gastrointestinal tract, from the oral cavity to the colon, though the terminal ileum is most commonly affected, while in UC they are restricted to the colon, spreading from distal to proximal and always involving the rectum [7,10–12].

The terminal ileum of patients with CD is characterized by transmural fibrosis and discontinuous inflammatory and fibrotic areas known as “skip lesions”. In disease-free ileal segments, TCs were found to exhibit a distribution comparable to healthy controls, whereas in disease-affected portions they were reported to be significantly reduced, especially in those areas with the most extensive fibrotic remodeling and severe intestinal wall architectural disruption [25,26]. In addition, in the muscularis propria of affected CD segments, the TC network appeared fragmented or even totally missing within both circular and longitudinal muscle layers, as well as around the myenteric plexus ganglia [25].

In UC, where fibrotic changes are typically confined mostly to the mucosal and submucosal layers of the colon, TCs were found to be significantly reduced even in early stages of the disease [27]. Such TC reduction closely paralleled colon fibrotic changes, as TC depletion was initially restricted to the muscularis mucosae and submucosa, while the muscularis propria, typically spared from fibrosis, remained unaffected, while in more advanced stages, TC loss also extended to deeper muscle layers and the myenteric plexus [27]. Interestingly, these findings align with those reported in “skip lesions” of disease-affected CD specimens, in which TC loss is found only in areas with pronounced fibrosis and architectural damage [27].

Notably, the reduction in TC numbers in both CD and UC was paralleled by a significant decline in interstitial cells of Cajal, which are considered the principal mediators of gut neurotransmission and motility and with which TC telopodes form intermingling networks in order to coordinate smooth muscle cell peristaltic activity [25,27]. The structural and functional impairment of this complex interstitial cell network at the myenteric plexus was thus supposed to account for IBD-associated gastrointestinal dysmotility [12,24,25,27].

Similar to dermal fibrosis, even in IBD the impairment of the intestinal network of TCs might be due to their entrapment in the fibrotic ECM, with consequent cell sufferance and significant alterations in the spatial relationships between telopodes and neighboring cells such as tissue-resident fibroblasts, thus favoring their uncontrolled activation and profibrotic transition into myofibroblasts [10,11,25,27]. Interestingly, although it has been suggested that TCs may also directly contribute to the increase in α-SMA+ myofibroblast population by transdifferentiating themselves and changing their immunophenotype [28], CD34/α-SMA double immunostaining failed to detect significant numbers of transitioning cells, which is in favor of a scenario in which TC degeneration predominates over transdifferentiation into myofibroblasts [10,11].

Finally, a recent study investigated TC distribution in tissue specimens from subjects affected by oral submucous fibrosis, a premalignant condition characterized by epithelial atrophy and progressive submucosal fibrosis [29]. In particular, the authors’ quantitative analysis of CD34+ stromal cells revealed a considerable decrease in TC number in the early stages of the disease, with a further decline in the advanced stages, prompting them to suggest the loss of TCs may contribute to the development of oral submucous fibrosis [29].

Although TC transplantation has not yet been explored as a possible treatment strategy for gastrointestinal pathologies, intestinal TCs exhibit considerable therapeutic potential as nonepithelial sources of Wnt ligands and R-Spondin 3 (RSPO3), two important regulators of the Wnt signaling cascade, which is known to be essential for intestinal stem cell self-renewal and proliferation within the gastrointestinal epithelium [23,30,31]. Indeed, conditional knockdown of Porcupine or Wntless genes, which are required for functional maturation of all Wnt proteins, in intestinal TCs led to a rapid interruption of Wnt signaling, a significant reduction of the stem cell population, and the consequent intestinal crypt collapse, underscoring the role of TCs as central mediators of intestinal homeostasis [23,30,31]. Furthermore, by using single-cell RNA sequencing, Kinchen *et al.* have identified a mesenchymal cell population located adjacent to the colonic crypt niche and characterized by a high Wnt signaling activity [32]. Interestingly, the decline of this population, presumed to be functionally analogous to TCs, was associated with the disruption of the intestinal epithelial architecture in UC, further supporting the notion that restoring TC function could be a promising therapeutic strategy for IBD [32].

## TELOCYTES IN LIVER AND BILIARY TRACT FIBROSIS

Liver fibrosis, primarily resulting from chronic hepatic injury due to viral hepatitis and alcoholic or nonalcoholic steatohepatitis, is characterized by excessive activation of hepatic stellate cells (HSCs) and abnormal ECM deposition leading to progressive disruption of liver parenchymal structures [7,10,11]. Hepatic TCs, which are predominantly located in the space of Disse, where they provide structural support to HSCs, hepatocytes, and stem cells, and are thought to contribute to the integrity of the organ architecture and cellular interactions [33], have been shown to undergo a significant depletion during hepatic fibrogenesis [34]. This finding, along with the concomitant increase in HSCs in fibrotic liver tissue, supports the notion that TCs constitute a distinct interstitial cell population within the hepatic stroma and that their reduction may contribute to HSC dysregulation, possibly fostering their profibrotic function [34]. In addition, since in a murine model of partial hepatectomy TCs have been demonstrated to exhibit close spatial proximity to both hepatocytes and hepatic progenitor cells, which are critical for liver regeneration, their disappearance in the context of liver fibrosis has been supposed to potentially lead to impaired hepatocyte function and stem cell niches compromise, further exacerbating the hepatic injury [35]. Interestingly, in the same study the authors reported that the posthepatectomy raise in hepatocyte proliferation coincided with a peak in both TC and hepatic stem cell numbers, suggesting a potential role for TCs in supporting hepatocyte and stem cell-driven liver regeneration [35]. Finally, TC displaying ultrastructural damages have been observed in a rat model of aflatoxin B1-induced liver injury [36].

As far as the biliary system, where TCs are broadly distributed within the muscular layer and are believed to contribute to biliary motility through electrical coupling with smooth muscle cells, histopathologic analyses on tissues affected by gallbladder stone disease and biliary dilation syndrome were performed to explore the possible relationship between TCs and such biliary disorders [37]. In particular, through immunohistochemical comparative approaches, the authors described a significant TC reduction in the fibrotic regions of both diseased gallbladder and bile duct compared to healthy control tissues, suggesting that such a decrease may disrupt the TC regulatory balance over fibroblast/myofibroblast activity, ultimately resulting into ECM alterations and progressive tissue fibrosis [37].

## TELOCYTES IN CARDIAC FIBROSIS

Within the human heart, TCs have been described in multiple anatomical compartments, including the myocardial interstitium, endocardium, epicardial stem cell niches, and cardiac valves [10,11,38]. Despite constituting a minor subset of cardiac interstitial cells, through their telopodes TCs are known to form an extensive 3D network in close association with cardiomyocytes, with which they interact via specialized junctions potentially facilitating electrical coupling and contributing to the formation of functional cellular units [10,11,38]. In epicardial stem cell niches, TCs have been found in intimate contact with resident cardiac stem cells, putative cardiomyocyte progenitors, as well as capillaries, nerve endings, and other stromal components [10,11,38,39]. This spatial organization suggests that TCs may play a critical role in maintaining a supportive microenvironment conducive to stem cell differentiation and maturation, thereby promoting myocardial regeneration [10,11,38,39]. Of note, the results of experimental studies in murine models further support a developmental role for TCs, implicating them in the regulation of myocardial compaction during embryogenesis [10,11]. Moreover, the shared expression of surface markers such as c-kit/CD117 between cardiac TCs and epicardium-derived progenitor cells has led to the hypothesis that TCs may also represent a specialized subpopulation within the cardiac progenitor cell hierarchy, with potential implications for both cardiac development and regenerative processes [10,11].

TC depletion has been documented across a wide spectrum of cardiac disorders [10,11,38,39]. During heart failure associated with dilated, ischemic, or inflammatory cardiomyopathies, TCs have been reported to be significantly reduced, with their number correlating with the extent and severity of alterations in ECM composition [10,11,38,40]. In particular, the TC network was found to be markedly diminished or even totally absent in the fibrotic regions of the failing myocardium, characterized by densely packed fibrillar collagen, with TCs exhibiting severe ultrastructural degenerations including cytoplasmic vacuolization and telopodal retraction [10,11,40]. Conversely, TCs appeared relatively preserved in myocardial regions rich in amorphous ECM components, where telopodes were indeed still elongated and displayed their typical branched morphology [10,11,40]. Interestingly, quantitative analyses revealed an inverse correlation between TC/telopode abundance and mature fibrillar collagen content, as well as a positive association with collagen degradation products [10,11,40]. These observations, supported by further in vitro studies demonstrating that ECM components can influence telopode behavior in cardiac TC cultures, collectively indicate that TC dynamics are closely linked to ECM composition and remodeling [10,11]. Though the full spectrum of pathophysiologic consequences resulting from TC loss remains to be elucidated, it has been postulated that their depletion may disrupt myocardial structural integrity and intercellular communications, ultimately impairing the spatial organization and signaling networks within the myocardium [10,11,40]. Specifically, the reduction of TCs may compromise the function and maintenance of cardiac stem cell niches, thereby limiting the pool of cardiomyocyte progenitors and impeding regenerative responses [10,11,40]. Of note, a significant TC loss was additionally documented in fibrotic areas of experimental rat models of myocardial infarction, further underscoring their sensitivity to fibrotic remodeling [10,11,17]. In the context of SSc, the fibrotic remodeling of the myocardium is similarly associated with a substantial reduction in the TC network, which may impair regenerative capacity and cardiomyocyte electrical coupling, thereby contributing to arrhythmogenesis and progressive heart failure [10,11,41].

Several preclinical studies have demonstrated the therapeutic potential of TCs in the heart [7,10–12,15^▪^,^17^,^42^^▪▪^]. In a rat model of myocardial infarction, intramyocardial injection of cardiac CD34+/c-kit+ TCs into the infarcted and peri-infarcted regions of the animals led to a substantial reduction in infarct size and improved postinfarction cardiac functions [17,42^▪▪^]. Since these beneficial effects were attributed to the reconstruction of the TC interstitial network and to the attenuation of myocardial fibrosis, restoring the TC network in infarcted myocardium may contribute to functional cardiac repair [17,42^▪▪^]. More recently, by using the same animal model, Liao *et al.* demonstrated that the administration of miR-21-5p, the most abundant microRNA identified in TC-derived exosomes, was able to reduce infarct size and fibrosis, improve cardiac function, and enhance angiogenesis [15^▪^]. These findings suggest that both TC-based and TC-derived exosome-based therapeutic approaches may hold promise for the treatment of ischemic heart disease and consequent myocardial fibrosis.

## TELOCYTES IN PULMONARY FIBROSIS

Pulmonary TCs, located within the interstitial spaces of intralobular bronchioles, terminal and respiratory bronchioles, and alveolar ducts, have been found to be significantly reduced in SSc-associated interstitial lung disease, possibly contributing to the progressive thickening of the alveolar septa and airspace obliteration [41]. Interestingly, lung TCs exhibit distinct gene and protein expression profiles that distinguish them from other mesenchymal cell populations [7,43,44]. Transcriptomic analyses have identified a set of genes selectively regulated in TCs [7,44]. Among the most upregulated are *FHL2*, a gene associated with anti-inflammatory responses and attenuation of fibrotic pathways, and *QSOX1*, which is involved in cell cycle regulation and ECM remodeling. Conversely, *PDE5A*, a gene whose overexpression is linked to the progression of pulmonary fibrosis, was found to be among the most downregulated in TCs [7,44]. These molecular signatures suggest a potential protective role for TCs in modulating both inflammation and fibrosis in pulmonary diseases [7,44].

The therapeutic potential of TCs against pulmonary fibrosis has been recently reported in an in vitro study by Zhang *et al.*, where the authors demonstrated that TCs were able to attenuate pulmonary fibrosis by preventing the epithelial-to-mesenchymal transition (EMT) in rat tracheal epithelial cells treated with TGFβ, mainly through the paracrine release of hepatocyte growth factor (HGF) [45]. Moreover, in a rat model of lipopolysaccharide-induced lung acute injury, which exhibits diffuse alveolar damage with interstitial fibrosis as its clinical hallmark, co-transplantation of CD34+ TCs and mesenchymal stem cells resulted in significantly reduced lung injury scores, supporting a synergistic therapeutic effect [46].

## TELOCYTES IN KIDNEY AND URINARY TRACT FIBROSIS

In the urinary system, TCs are primarily located in the renal cortical interstitium, particularly in the periglomerular and pericapillary regions, adjacent to Bowman's capsule, as well as within the upper lamina propria of the renal pelvis, ureter, and urinary bladder [7,47].

Loss or structural damage of TCs was reported to be closely associated with the occurrence of ureteral wall fibrosis. In a study by Wolnicki *et al.* a significant reduction in TC density and a concomitant increase in collagen deposition have been described in the thickened ureteral wall of pediatric patients with hydronephrosis, a prevalent condition affecting fetuses and neonates [48]. Similarly, in patients diagnosed with ureteropelvic junction obstruction, a decreased number of TCs has been correlated with an elevated collagen/muscle content ratio, indicating disrupted tissue architecture and fibrotic remodeling [49]. By combining immunohistochemical and double immunofluorescence staining with TEM techniques, Valente *et al.* also identified a significant increase in α-SMA expressing TCs in diabetic renal samples exhibiting periglomerular fibrosis [47]. Under TEM these TCs also exhibited ultrastructural changes such as dilated rough endoplasmic reticulum and electron-transparent niches containing proteoglycans, features suggestive of a synthetic, ECM-modifying phenotype [47]. On these bases, the authors suggested a potential role of TCs in the pathogenesis of diabetic nephropathy, possibly through their direct involvement in ECM composition regulation and fibrotic progression [47].

As far as their possible therapeutic potential, the in vivo administration of renal TCs in a model of renal ischemia-reperfusion injury mitigated histopathologic damages and improved renal function [50]. These protective effects were attributed to the paracrine activity of TCs, including the secretion of growth factors able to promote proliferation and inhibit apoptosis in renal tubular epithelial cells [50]. Moreover, in a rat model of unilateral ureteral obstruction-induced renal fibrosis, tail vein administration of cultured TCs was shown to upregulate HGF expression, inhibit EMT, suppress the TGFβ1/Smad signaling pathway, and ultimately attenuate the progression of fibrosis [18]. However, in vitro studies using a TGFβ1-induced fibrosis model failed to demonstrate any significant impact of TCs on EMT inhibition or HGF expression, suggesting that multiple in vivo interactions and environmental cues may be essential for TCs to be able to exert their antifibrotic effects [18]. Nonetheless, collectively these results support the notion that TCs may offer a novel therapeutic avenue for the treatment of renal fibrosis [18].

## TELOCYTES IN REPRODUCTIVE ORGAN FIBROSIS

TCs have been identified throughout the female reproductive system (i.e., vagina, uterus, fallopian tubes, ovaries, mammary glands and placenta), where many reports pointed out at their potential role in the regulation of local microenvironment, myogenic contractile mechanism, immunomodulation, bioelectrical signaling, and regulation of blood flow [51]. Additional studies also reported that TCs express steroid hormone receptors, suggesting that they might act as hormonal sensors, particularly in the context of reproductive physiology and pregnancy maintenance [51]. For instance, the ciliary beating frequency within the fallopian tube varies indeed in response to cyclical fluctuations of estrogen and progesterone, with low levels estrogen enhancing ciliary activity frequency, and high progesterone levels suppressing it [52].

Gynecological disorders such as premature ovarian failure, acute salpingitis, endometriosis, intrauterine adhesions, uterine leiomyoma, and ectopic pregnancy, are commonly associated with pathologic fibrosis [7]. In a murine model of cyclophosphamide-induced premature ovarian failure, apoptotic degeneration of ovarian parenchymal cells was observed in conjunction with a decrease in TC density in the fibrotic stroma, a phenomenon that was associated with lower estrogen levels and consequent disruption of the ovarian microenvironment and functionality [53]. Moreover, in rat oviduct tissues affected by endometriosis or acute salpingitis, TCs displayed severe ultrastructural abnormalities including organelle loss, nuclear and mitochondrial swelling, cytoplasmic vacuolization, endoplasmic reticulum dilation, and distension of intercellular junctions. These pathologic features led to the collapse of the interstitial TC network and the disruption of TC-stem cell niches, ultimately contributing to oviductal fibrosis and infertility [54,55]. Comparable findings were reported in human fallopian tube specimens from patients with endometriosis and tubal ectopic pregnancy, where damage and loss of TCs were associated with fibrotic remodeling and impaired tubal motility [56]. Further evidence of TC involvement in reproductive pathology comes from placental tissue in preeclamptic pregnancies [57]. Combined immunohistochemical and ultrastructural analyses, indeed, revealed a significant loss and morphologic abnormalities/degeneration of TCs within the fibrotic stroma of placental villi [57]. Similarly, a marked reduction in the c-kit+ TC population, with loss of TC-mediated homeostatic control and subsequent fibrotic development, was found in human samples of uterine leiomyoma, a benign myometrial tumor characterized by ECM overproduction [58]. In contrast to these findings, Karasu *et al.* reported an increased number of TCs in the muscular layer and serosa of tubal tissues from patients with ectopic pregnancy [59]. This was hypothesized to enhance the progesterone-mediated inhibition of tubal motility, potentially impairing the transport of the blastocyst to the uterine cavity [59]. A significantly higher number of TCs predominantly expressing progesterone receptors was similarly described in oviductal tissue samples from patients with uterine myoma, prompting the authors to propose that TC disturbance may contribute to infertility through both direct and indirect effects on smooth muscle contractility and ciliary motility within the oviduct [52]. Furthermore, recent studies in mammary gland tissue have identified TCs as potential precursors of cancer-associated fibroblasts in invasive lobular breast carcinoma, where they are thought to support tumor progression through ECM remodeling, promotion of tumor growth, invasion, and metastatic spread [60].

In the male reproductive system, TCs are widely distributed throughout the internal genital organs, including the prostate, testes, epididymis, and seminal vesicles [61–64]. In particular, in the testes TCs have been found to form an extensive reticular network within both peritubular and intertubular stromal compartments, where they are believed to contribute to testicular morphogenesis and homeostasis, as well as to the regulation of spermatogenesis and androgen secretion [65]. In tissue sections of seminoma, one of the most frequent malignant testicular cancers in humans, Marini *et al.* reported a significant TC depletion accompanied by a severe disription of the normal testicular architecture and the presence of interstitial fibrosis [65]. Interestingly, TC disappearance coincided with an expansion of α-SMA+ myoid cells, which may facilitate tumor progression and metastasis [65].

Notably, TCs from organs of the reproductive system have been shown to express and secrete matrix metalloproteinase-9, which may suggest a role of these stromal cells in ECM degradation and remodeling which are profoundly disturbed during tissue fibrogenesis [63,66].

Regarding the emerging therapeutic roles of TCs in the context of genital system disorders, initial evidence indicated that TCs enhance the in vitro proliferation, adhesion, and motility of endometrial stromal cells (ESCs), processes implicated in the pathogenesis of endometriosis [67]. Nevertheless, more recent studies have demonstrated that TCs may also promote in vitro decidualization by inducing mesenchymal-to-epithelial transition (MET) in ESCs via Wnt/β-catenin signaling pathway, which suggests a potential therapeutic application of TCs for reproductive disorders associated with impaired decidualization [68]. Indeed, proper decidualization and MET are fundamental for the cyclic renewal and regeneration of the endometrium, facilitating embryo implantation and regulating trophoblast invasion [68]. Finally, in a murine model of lipopolysaccharide-induced intrauterine adhesions and endometrial fibrosis, treatment with TC-derived exosomes was shown to reduce uterine fibrosis and enhance MET [16^▪^]. In addition, in vitro administration of either TC-conditioned medium or TC-derived exosomes inhibited TGFβ1-induced profibrotic differentiation of ESCs by providing a source of Wnts, as testified by a significant reduction in the expression of α-SMA, COL1A1, and fibronectin, an effect that was blocked in the presence of Wnt/β-catenin signaling inhibitors [16^▪^]. Taken together, these experimental findings further support the regenerative potential of TCs in uterine pathology.

## TELOCYTES IN CORNEAL FIBROSIS

By using an integrated immunohistochemical and TEM approach, CD34+/PDGFRα+ TCs have been identified throughout the full thickness of the corneal stroma, where they have been described to be aligned parallel to the corneal surface and interspersed among the ECM lamellae [69]. This regular spatial organization was hypothesized to contribute to the proper assembly and maintenance of the highly organized collagenous matrix, which is essential for ensuring corneal transparency and mechanical stability [69]. Notably, in the same study the authors also reported the existence of distinct TC subpopulations based on the co-expression of the stem cell marker c-kit/CD117, distinguishing between c-kit+ and c-kit– TC subpopulations [69]. Moreover, a comparative analysis between healthy corneas and corneas affected by keratoconus, a condition that leads to corneal fibrosis with disease progression, revealed a significant reduction in TC density within the pathologic tissues, particularly of the c-kit+ TC subset that likely represents a pool of progenitor cells with regenerative functions [69]. Of note, most of the remaining TCs in keratoconic corneas exhibited pronounced ultrastructural abnormalities, including organelle loss, cytoplasmic vacuolization, and telopode shrinkage or shortening, indicating not only a quantitative loss of these cells, but also a functional TC impairment during pathologic remodeling of corneal stroma [69].

## CONCLUSION

**FIGURE 3:**
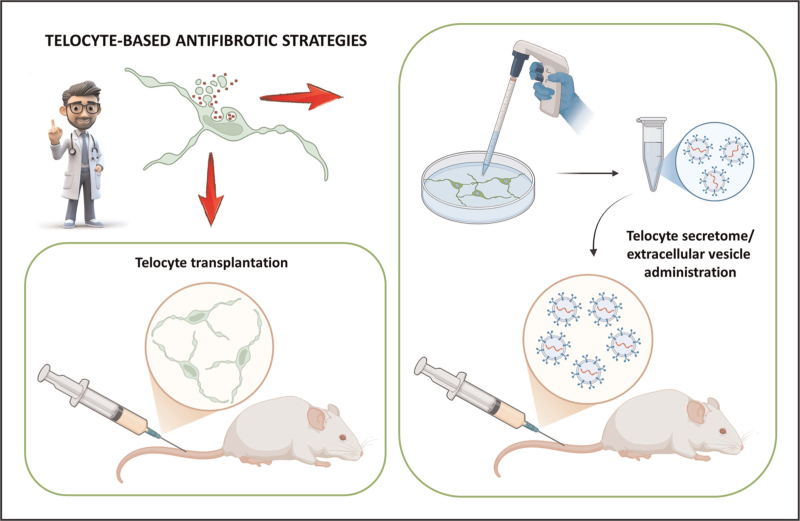
Potential telocyte-based antifibrotic strategies. Several preclinical studies demonstrated that telocyte transplantation or administration of telocyte-derived secretome/extracellular vesicles can attenuate tissue fibrosis and restore normal tissue architecture, supporting their potential as novel antifibrotic therapeutic tools.

Compelling evidence accumulated over the last decade has highlighted TCs as a distinct and functionally relevant stromal cell population with crucial roles in maintaining tissue architecture, mediating intercellular signaling, and supporting local stem cell niche renewal and regenerative properties [1–8,9^▪▪^,^10^,^11^]. Across a wide range of fibrotic disorders including SSc, IBD, liver and cardiac fibrosis, and fibrotic conditions affecting the reproductive, urinary, respiratory systems and the cornea, progressive loss or structural impairment of the TC network consistently emerged as a pathologic hallmark [4,6, 7,10–14]. While the causative relationship between TC dysfunction and tissue fibrosis remains to be fully elucidated, current data suggest that TC depletion may represent either a trigger or a consequence of aberrant fibrotic cascades, primarily through the loss of homeostatic regulation over fibroblast activation, and ECM turnover [4,6,7,10–14]. Notably, in vitro and in vivo preclinical models have demonstrated that TC transplantation and/or administration of TC-derived secretome or exosomes can mitigate fibrogenesis, restore tissue architecture, and improve organ function, thereby underscoring their therapeutic potential (Fig. 3) [9^▪▪^,^12^,15^▪^,16^▪^,^17^,^18^,^42^^▪▪^]. Future research should aim to clarify the molecular mechanisms underlying TC-mediated antifibrotic effects, define their cell-to-cell and paracrine interactions with fibroblasts, immune cells and stem/progenitor cell niches, and explore novel strategies for their isolation, expansion, and delivery for therapeutic purposes. As tissue fibrosis remains a major unmet clinical challenge, TCs represent a new promising cellular target for the development of innovative antifibrotic therapies.

## Acknowledgements

*All the figures in this paper were created in part with BioRender.com.*

### Financial support and sponsorship

*None.*

### Conflicts of interest

*There are no conflicts of interest.*

## REFERENCES AND RECOMMENDED READING

Papers of particular interest, published within the annual period of review, have been highlighted as:

▪ of special interest
▪▪ of outstanding interest
